# History of the Life of D. Hayes Agnew, M. D., LL. D.

**Published:** 1893-06

**Authors:** 


History of the Life of D. Hayes Agnew, M.D., LL.D. By J. Howe Adams, M. D. With fourteen full-page portraits and other illustrations. In one large royal octovo volume, 376 pages, extra cloth, beveled edges, $2.50 net; half-morocco, gilt top, $3.50 net. *Sold only by subscription.* Philadelphia: The F. A. Davis Co., Publishers, 1914 and 1916 Cherry Street.

The life of Dr. Agnew by Dr. J. Howe Adams is a fitting example of a worthy subject treated by a most able author. The work is enriched by various portraits of the learned doctor at different stages of his life, and as the biographies of celebrated men of the times are the history of a country, so in giving the life of the noted physician its author has given valuable historical information of our city and of its noted medical schools, the University of Pennsylvania and the Jefferson College, his life-work was so dedicated to medical science that he has become part of their history.

The first chapter of the book is devoted to the lineage of the Agnew family, tracing it back to its earliest records. The name is derived from Agneaux, a quaint little village in Northern France. The author then tells of his business ventures, of his founding the School of Anatomy, also of his success as a writer. Chapter X is devoted to the Garfield case, another leaf in the country's history. Then follows Dr. Agnew's home life and finally his death.

The book is enriched by a chapter from the pen of our most noted physician and Provost of the University of Pennsylvania, Dr. William Pepper, a gem worthy of its well-adapted mounting. The volume will prove encouraging and useful to the young physician starting on the road of life, who, by this example, will see that success is only gained after years of patient and faithful work; and to his contemporaries by the pleasing recollections it will bring of familiar names. Read the work, it will well repay the time expended.

